# Yoga Training in Junior Primary School-Aged Children Has an Impact on Physical Self-Perceptions and Problem-Related Behavior

**DOI:** 10.3389/fpsyg.2016.00203

**Published:** 2016-02-23

**Authors:** Stefanie Richter, Maike Tietjens, Susanne Ziereis, Sydney Querfurth, Petra Jansen

**Affiliations:** ^1^Institute of Sport Science, University of RegensburgRegensburg, Germany; ^2^Institute of Sport and Exercise Sciences, University of MuensterMuenster, Germany

**Keywords:** yoga, physical self-concept, anxiety, executive function, motor function, children

## Abstract

The present pilot study investigated the effects of yoga training, as compared to physical skill training, on motor and executive function, physical self-concept, and anxiety-related behavior in junior primary school-aged children. Twenty-four participants with a mean age of 8.4 (±1.4) years completed either yoga or physical skill training twice a week for 6 weeks outside of regular school class time. Both forms of training were delivered in an individualized and child-oriented manner. The type of training did not result in any significant differences in movement and executive function outcomes. In terms of physical self-concept, significant group differences were revealed only for perceived movement speed such that yoga training resulted in perceptions of being slower while physical skill training resulted in perceptions of moving faster. Analysis of anxiety related outcomes revealed significant group effects only for avoidance behavior and coping strategies. Avoidance behavior increased following yoga training, but decreased following physical skill training. In addition, following yoga training, children showed an increased use of divergent coping strategies when facing problematic situations while after physical skill training children demonstrated a decrease in use of divergent coping strategies. Changes in overall physical self-concept scores were not significantly correlated with changes in avoidance behavior following yoga training. In contrast, following physical skill training increased physical self-concept was significantly correlated with decreases in avoidance behavior. In sum, exposure to yoga or physical skill training appears to result in distinct effects for specific domains of physical self-concept and anxiety-related behavior. Further studies with larger samples and more rigorous methodologies are required to further investigate the effects reported here. With respect to future studies, we address potential research questions and specific features associated with the investigation of the effects of yoga in a sample of school-aged children.

## Introduction

Yoga is a traditional technique to improve health and wellbeing by way of exercises, breathing, and meditation (for detailed information on the roots of yoga see, for example, Gard et al., 2014). In recent years, the interest in yoga as an alternative medicine intervention, but also as a means to prevent diseases and foster normal functioning and development has increased. At the same time, there is an increasing effort to scientifically establish the positive effects of yoga on adult’s and children’s motor function, emotion and cognition (Galantino et al., 2008; Kaley-Isley et al., 2010; Balasubramaniam et al., 2013). Changes in motor function may, in turn, modify the physical aspect of adults’ (Moore et al., 2011; Musanti, 2012) and children’s self-concept (Ekeland et al., 2005). In the present pilot study we asked whether yoga training in primary school-aged children has an effect on body, emotion, and cognition. To our knowledge, there is no study analyzing the effects of yoga in its entity in this age group.

Asanas, mostly static body positions, are an integral part of the yoga-practice. The effect of yoga on (static) motor function was investigated in two studies by Telles et al. (1993, 1994). They observed improvements in hand steadiness after yoga training in young adults (Telles et al., 1994) and children (9–13 years; Telles et al., 1993). Beneficial effects of yoga training on handgrip strength and handgrip endurance–as a measure of force fluctuation during isometric contraction–were shown for adults (Thangavel et al., 2014) and adolescents (Mandanmohan et al., 2003). However, no changes in handgrip strength were found in a study by Tracy and Hart (2013) in young adults. Differences in statistical approach may account for the diverging results. Whereas the workgroups of Telles, Mandanmohan, and Thangavel computed within group pre-post comparisons (Wilcoxon signed rank test, paired *t*-test) and asked whether the *p*-values were significant in the yoga group only, in the study of Tracy and Hart, a two-way ANOVA was calculated with both groups included in one and the same statistical analysis. Next to static motor functions, yoga draws upon accurate motor performance. Accordingly, adult subjects of a yoga group outperformed subjects of a waiting control group (i.e., a control group with no intervention whatsoever) in a mirror-tracing task resorting to eye-hand coordination, as well as motor speed and accuracy (Telles et al., 2006). The results of Telles et al. (1993, 1994, 2006), Mandanmohan et al. (2003), and Thangavel et al. (2014) suggest that yoga training may improve relevant variables in motor function both of adults and older children. However, the effects may not be as strong as to withstand stricter statistical approaches (Tracy and Hart, 2013). What we do not know is whether yoga training improves motor abilities in younger children.

Second, the so-called EXEM-model by Sonstroem and Morgan (1989) supposes that the physical self-concept is part of the general self-concept, a structured description of the self, which is accessible to consciousness. Self-esteem is the evaluative component of the self-concept. Changes in physical self-concept (and, consequently, in general self-concept) are hypothesized to be due to changes in perceived physical competence, i.e., an evaluation of the self with respect to physical skills and fitness. In their review, Babic et al. (2014) conclude that physical activity and physical self-concept are closely linked. Corresponding correlation studies are found both for adults (e.g., Lindwall and Hassmen, 2004), older children (10–14 years; Crocker et al., 2000), and younger children (6 years; Planinsec and Fosnaric, 2005).

With respect to adults, beneficial effects of sports’ interventions (resistance exercise and aerobic training) on physical self-concept were found in the studies by Moore et al. (2011) and Musanti (2012) in healthy subjects and women surviving breast cancer, respectively. In children with a mean age of 11 years, Mayorga-Vega et al. (2012) found that fitness-increases were not accompanied by changes in the physical self-concept. Apart from the fact that the samples differed, Mayorga-Vega et al. (2012) investigated effects of an 8-weeks physical fitness program with individual sessions lasting only minutes. Thus, resistance exercise, but also aerobic training led to an increase in physical self-concept, but in older children, no effects were found with a small physical fitness program.

The results of Moore et al. (2011), Mayorga-Vega et al. (2012), and Musanti (2012) refer to physical activity and its impact on physical self-concept. The effects of yoga were studied mainly in relation to the broader dimension of general self-esteem. In a study by Kovačič and Kovačič (2011), self-esteem increased in a yoga group in women after breast cancer surgery. Similar results for healthy subjects were found in a study by Taspinar et al. (2014). However, benefits for self-esteem were not only found with yoga, but also with other kinds of training in the studies by Muller et al. (2006) and Deshpande et al. (2008). In children, the picture is also mixed. In a study by Telles et al. (2013), general self-esteem increased in a yoga group, but not in a physical activity group in children aged 8–13 years. In contrast, in a study by White (2012), self-esteem increased in a yoga and a control group with mindfulness training in fourth- and fifth-grade girls. Two more studies showed a positive effect of yoga in 15 year-old children, however, there were no control groups (Conboy et al., 2013; Sethi et al., 2013).

Thus, there seem to be potentially positive effects of physical activity on the physical self-concept in adults and adolescents, and of yoga on general self-esteem in adults and older children. However, other forms of activity also seem to have an effect. Data concerning the relation between yoga and physical self-concept are missing in all age groups. It is of importance to find out if increases in physical self-concept may be induced by way of yoga-training, as increases in physical self-concept could improve general self-concept and self-esteem (as suggested by the EXEM-model).

Third, in two studies with adult patients, positive effects of a yoga training on anxiety have been found (Yadav et al., 2012; Köhn et al., 2013). With respect to healthy subjects, a beneficial effect of yoga was also found in a study by Yoshihara et al. (2014), however, there was no control group in this study. In the study by West et al. (2004), both African dance and Hatha yoga reduced perceived stress and negative affect in a sample of healthy college students. Thus, stress reduction in adults may not only be achieved by means of yoga. Platania-Solazzo et al. (1992) showed that a relaxation therapy including yoga reduced anxiety in children and adolescent psychiatric patients. In a study by Stueck and Gloeckner (2005), yoga reduced fears and feelings of helplessness and increased emotional balance in fifth-graders. In sum, there is evidence showing a positive effect of yoga on healthy adults or adults and children with physical, emotional or psychiatric dysfunctions. However, other forms of activity have also proven effective in adults. The situation with healthy children is less clear. Does yoga have a positive effect on anxiety in this subject group compared to other forms of physical activity?

Fourth, yoga has been shown to have a positive effect on cognition. Advantages of a yoga training compared to breath awareness and exercise were found in the studies by Telles et al. (2007) and Rocha et al. (2012) for visual attention and memory, respectively. Short-term positive effects on math functions were found in a study by Field et al. (2010) with a combined Tai chi and yoga class. However, there was no control group in this study and it is not possible to disentangle the effects of yoga and that of Tai chi. No advantage of yoga was found in the study by Telles et al. (2012), where performance in a digit-letter substitution task increased in adults who performed yoga or breath awareness, but also in the control group who listened to meditation music.

In the study by Naveen et al. (1997), yoga breathing techniques resulted in increased spatial memory in children (10–17 years). Moreover, several studies have shown positive effects of yoga in children with attentional deficits or ADHD (e.g., Harrison et al., 2004; Haffner et al., 2006). However, there is also contradictory evidence. Peck et al. (2005) found no positive effect on ADHD-symptoms in a 3 weeks program with children doing yoga to a 30-min videotape twice a week. Maybe the training was not intense enough to show effects in this study. Regarding healthy children, increases in visual attention and concentration were found in 15-year-old girls from low income families after yoga training in a single-group study by Sethi et al. (2013). In the study by Chaya et al. (2012), no differences in cognitive performance (Indian adaptation of the Wechsler Intelligence Scale for Children) were found with socioeconomically disadvantaged primary school-aged children doing a yoga or physical activity training. Thus, both in adults and older children, some studies show a benefit of yoga compared to other forms of physical activity or treatment on cognition, but there are also contradictory results as well as studies, in which no conclusion can be drawn with regard to specific effects of yoga due to missing control groups.

Related to the research dealing with yoga and its effects on attention, math, or IQ are studies which focus on executive functions, specifically. Diamond and Lee (2011) identified these functions as one crucial factor for success in school. Core executive functions include cognitive flexibility, inhibition (self-control, self-regulation), and working memory (Miyake et al., 2000). Gothe et al. (2013) found acute effects of a yoga training on executive functions assessed immediately after the training in young females. No effects were found after aerobic exercise and in a baseline condition without any training. However, these differences between conditions were evident only for more difficult tasks. Positive effects of yoga training on executive functions were also found in a study by Manjunath and Telles (2001) in 10- to 13-year-old girls. Thus, with executive function, effects of yoga have been found in young women and older children; however, studies in younger children are still missing.

In sum, large parts of research in the field of yoga focuses on specific adult samples like patients (e.g., Yadav et al., 2012) or occupational groups (e.g., Rocha et al., 2012). With respect to children, mainly older age groups (e.g., Naveen et al., 1997; Mandanmohan et al., 2003) and children with a medical diagnosis (e.g., Platania-Solazzo et al., 1992; Haffner et al., 2006) or which are socioeconomically disadvantaged (e.g., Chaya et al., 2012) have been studied. The picture is even more complex due to the fact that some studies did not use control groups (e.g., Moore et al., 2011; Sethi et al., 2013) or performed statistical comparisons within yoga- and control groups, separately (e.g., Telles et al., 1993, 1994). Moreover, although differential effects may be expected with yoga due to its focus on relaxation and attention (Berger and Owen, 1988, 1992; Arnsten, 1998; Oken et al., 2006; Sharma et al., 2013), also other kinds of physical activities or treatments showed benefits regarding, for instance, physical self-concept or anxiety (e.g., West et al., 2004; Deshpande et al., 2008).

In younger children, data are generally missing. Considering the fact that already young, normally developing children react with stress to increases in performance requirements in school (Stueck and Gloeckner, 2005) it is important to find out if yoga can be introduced to aid normal functioning and development in a holistic manner. Therefore, in the present pilot study we investigated yoga vs. physical skill training in this age group and its effects on motor functions and physical self-concept, as well as emotional and cognitive functions. Regarding the latter, especially executive functions were considered because they play an important role in academic success. Physical skill training was chosen as a control activity, because it is part of the normal physical activity lessons in the school context.

We asked whether there are stronger effects of yoga as compared to physical skill training on emotion (anxiety) and cognition (attention, inhibition) due to a stronger focus on attention and relaxation/stress reduction in yoga than in other physical activities. Specifically, we assumed that yoga has an impact on emotional wellbeing by way of reducing stress or autonomic arousal in response to stressful events (Sharma et al., 2013). In addition, biological stress markers have been shown to decrease after yoga training (Platania-Solazzo et al., 1992; Kamei et al., 2000; Rocha et al., 2012). Effects of yoga on executive function may also be mediated by an enhancement in mood and a reduction of stress (Berger and Owen, 1988, 1992; Arnsten, 1998). Additionally, general improvements in attention may also play a role (Oken et al., 2006). With respect to motor function, previous results suggest that yoga has some beneficial effects on balance and hand skills. However, it is difficult to decide how different kinds of training may influence these skills, since these studies resorted to waiting control groups only. We suppose that effects are basically dependent on the kinds of skills addressed in the training. Finally, we supposed that yoga would have a greater impact on the physical self-concept due to a supposedly stronger focus on the perception of the self than physical skill training.

## Materials and Methods

### Subjects

The experiment was performed at a Catholic primary school in Muenster, Germany. Initially, 25 children were included in the study, however, one child was ill at the time of the posttest so that all in all, 24 children aged 6–11 years participated in the study (mean age: 8.4 ± 1.4 years, 12 boys, and girls, each).

The study was not approved by an institutional review board or equivalent committee as there were no negative physical or psychological consequences of the tests or training programs to be expected in the participating subjects. With respect to the anxiety questionnaire we applied and which might be considered a potential risk, detailed information is given below. In addition, there were two computer-based tests requiring button-presses (which may probably remind the children of some computer games), the movement-ABC, which is similar to what children know from physical education, and a physical self-concept questionnaire, which asks the children to assess their physical competence (please see below). All of these seem inoffensive to us. With respect to the training, we would like to say that both trainers are experienced in working with primary school-aged children and both trainings were adjusted to the age range (please see below). We declare that our approach is in line with national and international human research ethics policies and that we have made clear and communicated all considerations necessary to assess the question of ethical legitimacy of the study.

In the school, written information material was distributed among pupils who could pass it to their parents. Parents and their children could decide at home if they would like to take part in the study and pupils could bring the signed consent back to school. In the written information material, parents were elaborately informed about all the tests applied to their children. Moreover, they were told that they could have a look at the test material (including the anxiety questionnaire), but none of the parents made use of this possibility. Thus, parents could register their child to the study by giving written informed consent in accordance with the Declaration of Helsinki. Parents and children were informed that participation is voluntary and could be finished at any time during the experiment. Children were assigned to a yoga and physical skill training group according to their time schedule. We are aware of the fact that this is criticizable, but as the training was performed in the scope of the full-time education over several weeks, we had to consider other occupations of the children during the afternoon. Mean age in the yoga group was 7.7 ± 0.9 years (seven boys, five girls); in the physical skill training, mean age was 9.1 ± 1.5 years (five boys, seven girls). Please note that for the physical skill training, post-test data of all 12 subjects were available only for the motor test. For the physical self-concept, the anxiety questionnaire, and the cognitive tests, data sets were available only for 10 children, since post data of two children got accidently lost. Moreover, the number of children that were included in the statistical analysis of the employed tests and questionnaires varied due to outliers. A detailed description of the numbers can be found at the end of the Methods section.

### Experimental Setup

The experiment was conducted between April 28, 2014, and June 27, 2014 (i.e., Easter and summer holidays). We used a pre-posttest-design with yoga training, respectively, physical skill training in between. In the experimental group, yoga training was conducted by a certified trainer; a graduate university student of sports science guided the physical skill training group’s training. This student had guided similar training groups in schools before, i.e., was experienced with this regard. Training was performed twice a week for 45 min over a total of 6 weeks. Pretest, training sessions and posttest were conducted primarily during the afternoon and outside of regular sports lessons in the scope of full-time-education (until 4:00 p.m.). The structure of testing sessions was identical for pre- and posttest. Each session started with some minutes of conversation between the child and the experimenter about everyday issues not related to the experiment, so that the child could adapt to the situation. We implemented tests of executive and motor functions, a questionnaire on the physical self-concept and an anxiety questionnaire in the denoted order: Flanker test (10 min), Go-Nogo test (15 min), physical self-concept questionnaire (10 min), anxiety questionnaire (20–30 min), and motor test (20 min). The physical self-concept questionnaire was administered ahead of the Movement-ABC 2 to get a measure that is not confounded by recent motor performance. Total duration of individual testing sessions was about one and a half hours. Up to four children were tested simultaneously at different desks in one of the school’s common rooms. The motor test was performed in the gym or the assembly hall and, due to organizational reasons, on the subsequent day to the rest of the tests and questionnaires.

### Measures

#### Tests of Executive Functions

##### Flanker test

**FIGURE 1:**
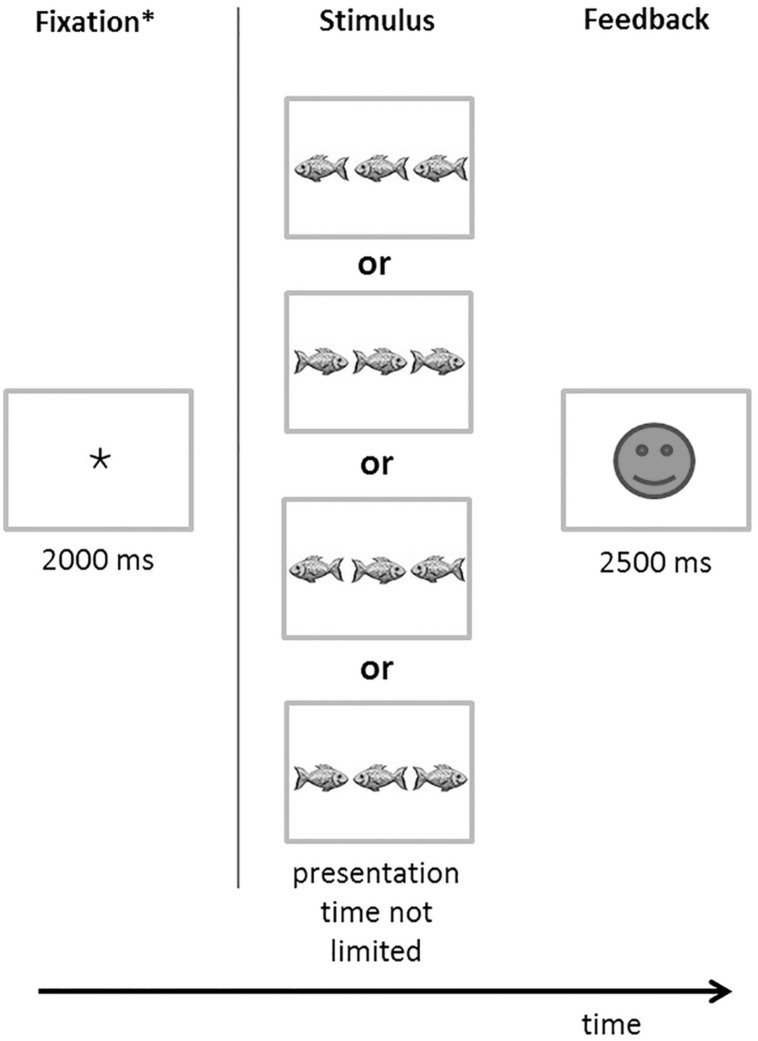
**Time line of trials in the Flanker test**. At the beginning of the test, a fixation cross was presented for 2000 ms. Afterwards, one of the four different stimuli was shown, until the child reacted with a button press. Finally, a sad or laughing smiley was presented for 2500 ms (dependent on the correctness of the response). ^∗^The next trial started with the presentation of the next stimulus, i.e., the fixation cross was not shown again.

The Flanker test (Eriksen and Eriksen, 1974) measures inhibition, i.e., a core component of executive functions. The aim of the test is to analyze how well children are able to not react to irrelevant stimuli. Children are shown, on a 15-inch laptop monitor, pictures of three fish (see **Figure 1**). The fish that is flanked by the two other fish “swims” either in the same or opposite direction of the neighboring fish. The child is asked to help feed the fish in the middle by indicating (with a button-press) the direction in which it is swimming. The task is more difficult, if the swimming direction of the three fish is not in agreement. The time line of each trial was as follows (see **Figure 1**): at the beginning of the test, a fixation cross was presented for 2000 ms. Afterward, one of the four different stimuli was shown, until the child reacted with a button press. Finally, a sad or laughing smiley was presented for 2500 ms (dependent on the correctness of the response). The next trial started with the presentation of the next stimulus, i.e., the fixation cross was not shown again. In total, there were 40 trials (same direction right: 10 trials, same direction left: 10, different direction, middle fish right: 10 trials, different direction, middle fish left: 10 trials). During trials, no feedback was given by the experimenter. We measured reaction times and errors in the different conditions.

##### Go-Nogo test

**FIGURE 2:**
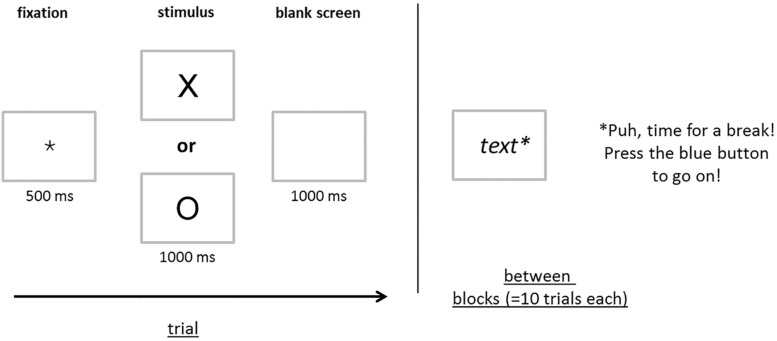
**Time line of trials in the Go-Nogo test**. First, a fixation cross was shown for 500 ms, followed by the cross or circle, which was presented for a maximum of 1000 ms. Thereafter, or after the child’s reaction, if earlier than 1000 ms, a blank screen was presented for 1000 ms. After 10 trials, a break was introduced by the text “Puh, time for a break! Press the blue button to go on!”

The Go-Nogo test by Drewe (1975) measures attention. On a 15-inch laptop monitor, the children are subsequently shown either a cross or a circle (see **Figure 2**) in random order. They are asked to react with a button press to the cross only. The time line of each trial was as follows (see **Figure 2**): first, a fixation cross was shown for 500 ms, followed by the cross or circle, which was presented for a maximum of 1000 ms. Thereafter (or after the child’s reaction, if earlier than 1000 ms), a blank screen was presented for 1000 ms. Then the next trial started with the presentation of the fixation cross. After 10 trials, a break was introduced by the text “Puh, time for a break! Press the blue button to go on!” Thus, the child could decide by him-or herself how long the break took. Reaction times and errors (false positive, omissions) were registered. There were 100 regular trials (70 crosses, 30 circles) and 15 practice trials (8 crosses, 7 circles). During the practice trials, children were given feedback by the computer program. If the child’s response was correct, a green check was presented, if it was incorrect, a red cross was shown (1500 ms; please note that practice trials are not shown in **Figure 2**). Furthermore, the experimenter complimented the children if they did well or encouraged them and gave further explanations if errors occurred. During regular trials, no feedback was given, neither by the program, nor the experimenter.

#### Physical Self-Concept Questionnaire for Children (PSC-C)

The PSC-C by Dreiskämper et al. (2015) is an adapted version of the PSDQ by Marsh et al. (1994) and its German translation by Stiller et al. (2004) for children aged 9–12 years. In the development of the PSC-C, 24 items were chosen from the 46 items of the test by Stiller et al. (2004) to adapt the questionnaire to the group of primary school-aged children. These items capture different aspects of a child’s belief concerning his or her motor skills and capacities. The questions are answered on a 4-point-scale ranging from totally disagree (one point) to totally agree (four points), and can be grouped into 7 motor categories (strength, endurance, speed, flexibility, coordination, appearance, sports competence). Examples of questions are: “I am strong” (strength), “I can run for a long time without getting tired” (endurance), “I am fast” (speed), “I am flexible” (flexibility), “I can easily control and guide my movements” (coordination), “I am content with my body” (appearance), “I am good at sports” (sports competence). Scores were calculated for each of the 7 subscales by summing up the points across answers. Dependent on the child’s reading capacities, the questions were read to the child or the child read them by his- or herself.

#### Anxiety Questionnaire (BAV 3–11)

The BAV 3–11 (“Bochumer Angstverfahren für Kinder im Vor- und Grundschulalter”, Mackowiak and Lengning, 2010) is a German questionnaire to assess anxiety in 3- to 11-year-old children. Usually, both children and their parents provide information about anxiety, typical physical reactions to anxiety, and preferred coping strategies. In the present study, however, only children’s ratings were used, as our aim was to find out if yoga improves the child’s feeling about his or her anxieties (there was no diagnostic approach, for which the parents’ ratings would have been indispensable).

In the BAV 3–11, children are presented with situations in the form of pictures that are verbally paraphrased by the experimenter. These situations might be frightening when encountered in reality. We decided to use the BAV 3–11 as it is a common German test of anxiety standardized in a sample of 1000 children. It is used in educational counseling, school-psychology, prevention, and educational and psychological practice and research. The test is actually an assessment of typical reactions to situations children know or which are at least not far from children’s experience: e.g., getting back a ball that has accidently been thrown in the neighbor’s garden, balancing over a tree trunk across a river, stay overnight with a friend, being confronted with a spider, going to the doctors, asking other children if one may play with them, say hello to an adult friend, having to walk past a dog, being alone at home, climbing a climbing frame, get lost in a wood. These situations are presented in a factual manner, so that the child may think about similar situations and how he or she would react or feel (or has reacted or felt in the past). So anxieties are not evoked in the test. One may argue that it cannot be anticipated if children have such a vivid imagination as to react with real fear to the pictures and their description. However, we felt that this is quite unlikely due to the fact that the situations were not highly detailed, e.g., by a story. Moreover, the BAV 3–11 is a frequently used inventory, which was actually developed for pre-school and primary school-aged children. The internal consistency for the whole version as well as the two parallel versions lies between α = 0.64 und 0.86. Construct validity was verified by factor analysis, criterion validity by means of correlations with other questionnaires and parents’ ratings. In addition, intra-individual changes were analyzed with repeated applications (after 1 ½ years).

The experimenter, who did not know which experimental group each child belonged to, noted the child’s reported anxiety, coping strategies, and physical reactions. All in all, 26 pictures were presented in about half an hour. The situations belonged to one out of three scales: social fears (seven situations, example: getting back a ball that has accidently been thrown in the neighbor’s garden), body-related fears (nine situations, example: balancing over a tree trunk across a river), cognitive fears, worries and apprehensions (eight situations, example: stay overnight with a friend), phobia (two situations, example: being confronted with a spider).

Reactions were determined on three levels, according to the instructions in the manual:

Subjective experience: children were asked: “How do you feel?” and gave their answer by pointing to a 5-point-scale of “smileys” (happy to frightened faces). If the child pointed to a frightened face, the answer was counted as one point, if the child pointed to a laughing smiley, 0 points were noted. The points given for each answer were summed up as a “general anxiety”-score (maximum score 26).

Behavioral level: children were asked: “What do you do?” and each answer was categorized according to the behavioral strategy described by the child. There were seven different types of strategies: (1) looking for information, (2) taking an action, (3a) looking for support and shelter, (3b) taking an action with support, (4a) inhibition of action, (4b) behavioral disorganization, (5) cognitive regulation, (6) flight/avoidance, (7a) mentioning phantasies, (7b) miscellaneous.

Several kinds of scores were calculated from the child’s responses: for the score “all strategies”, it was noted how often each kind of strategy was used, and these frequencies were summed up across all strategies. The strategies “looking for information” and “taking an action” (1, 2) were subsequently summarized under the regulation strategy “problem-oriented behavior”. The score was calculated as: (number of times strategies 1 and 2 are used/number of times all strategies are used) × 100. “Inhibition of action”, “behavioral disorganization”, and “flight/avoidance” (4a, 4b, 5) were summarized under the notion of “problem-avoiding behavior”. “Looking for support and shelter”, and “taking an action with support” (3a, 3b) made up the score “social support”. The calculation of the latter two scores was similar to that of “problem-oriented behavior”. A score named “different strategies” illustrates how many divergent strategies a child used. For each strategy it was noted, if it was used at least once with the 26 situations. If so, a value of 1 was given, otherwise 0. The numbers were summed up (maximum = 10 with the strategies 1, 2, 3a, 3b, 4a, 4b, 5, 6, 7a, 7b).

Physical level: children were asked: where do you feel the fear in your body?” and spontaneous reactions of the child were noted. If children had difficulties with this question, a draft of a child’s body was shown and the child was asked to point to the location where fear was felt (e.g., in the stomach as with stomach trouble, in the heart as in rapid heartbeat, in the head as with headache). Children were asked maximally twice throughout the whole test with the first mentioning of medium anxiety and/or with strong anxiety. If physical anxiety reactions occurred, they were scored with 1 point (otherwise 0 points). Thus, the maximum score was 2. Thus, if a child never reported a medium or strong anxiety as subjective experience, he or she was not asked about the physical level of his or her anxiety at all.

Finally, with the help of norm tables, all raw values can be converted in *T*-values. These *T*-values were entered into the statistical analysis of the present study.

### Movement-ABC 2

The Movement-ABC 2 (Petermann, 2008) is a common standardized test of fine and gross motor function in children aged 3–16 years. Depending on the age group (3–6 years, 7–10 years, 11–16 years), it comprises the following subtests (the respective task for the youngest group is described first, followed by the task for the medium-age group and that for the oldest group):

#### Hand Skills

Grab coins from the table and put it through a slot in a box; put pens in a board with openings; turn two-colored coins that are stuck in a board so that the other color points upward.

Bead plastic pearls on a lace; thread a lace through the holes of a plastic board; put plastic bars together to build a triangle with the help of screw and screw nut.

Draw a line between two parallel meandering borderlines on a sheet of paper as good as possible and without touching the borders (same task for all three groups).

#### Ball Skills

Catch a small bag of beans thrown by the experimenter; catch a tennis ball with two hands that the child has thrown to the wall; catch a tennis ball with one hand that the child has thrown to the wall.

Throw a small bag of beans in a target circle on a mat (youngest and medium age group); throw a tennis ball to the wall, targeting a red circle.

#### Balance

Stand on one leg for a certain amount of time; balancing on a board; balancing on a board while the heel of one foot touches the toes of the other foot (static balance).

Walk with lifted heels on a line; walk heel-to-toe on a line; walk heel-to-toe backwards on a line (dynamic balance).

Jump on both legs over a row of mats; jump on one leg over a row of mats; jump diagonally from one mat to the next (dynamic balance).

According to the test manual, we measured how long the child took to complete a task (as in task 1) or how long the child was able to perform a task (as in task 6). Moreover, errors were registered. Both, time and error measures were transformed to test scores according to the manual.

### Training

We payed special attention to a child-oriented character of the training, with little pressure exerted on the children and playful elements. Moreover, both trainings were individualized in nature, i.e., each child trained on his or her own. However, training was still performed in a group setting. Whereas yoga training was performed in a suitable room of the part of the school where pupils are taken care of in the afternoon, physical skill training took place in the school gym.

#### Training in the Yoga Group

At the beginning, children and trainer sat together in a circle to say hello. One after the other, each child got a gemstone to hold, which was the sign that it was his/her turn to tell how he/she felt today and what he/she had experienced since the last session. Afterward, participants stood up and shook their bodies to “shake off all the rage”; alternatively the whole group did the jumping jack. Then the actual yoga-practice followed, which was embedded in a story, like walking through the forest. The children were, for instance, encouraged to imagine a deer behind a small bush, that had observed the participants for some time. Then they imagined walking through soft dosh, little bugs crawling around the participant’s feet, hearing a loud buzzing from the bees that had built their nest on a tree. Next, the children had to imagine that the moon was already shining and that they had to go home. In this example, the associated asanas were: tree, sun, deer, bug, bee, and moon. At the end of the session, the participants stood together in a circle again, holding each other’s hand and reciting a slogan: “I stand like a tree and I have self-confidence” (which is actually a rhyme in German: “Ich steh’wie ein Baum und habe Selbstvertrauen”). Then the participants said goodbye. Thus, the asanas performed in the yoga training were connected by way of a story. This way, the trainer picked up children’s natural joy about stories to preserve their attention and concentration.

#### Training in the Physical Skill Training Group

First, children and trainer sat together to say hello and talk about what would follow in the training session, which was the so-called “movement landscape”. The main part of the session consisted of free play, with the trainer attending and assisting the children at different “stations” (see below). At the end of the session, the participants sat together again to reflect the training.

The stations of the movement landscape encompassed: balancing (over a “river” on a rope); swinging over the “grand canyon” (with rings and ropes); throwing and targeting with a ball; riding a course on a large “skateboard” (i.e., a board to sit on);“crevasse” (two soft mats were positioned behind a climbing frame with a gap in between; the children could try to get up the gap; some more mats were laid out for security reasons); walking in circles [a large mat (loosely rolled up) was set up vertically, and the children could walk within this spiral]; Bobbycar-race; Volleyball (“throw the ball over the magic string”); bobsleigh run (two benches were leant against a box to build a slide, two benches were positioned right and left of it as “fence”; at the end of the slide there was a soft map to stop the slide); “Matterhorn” (rope climbing).

Taken together, one may note both similarities and differences between the two groups. First, both trainings demanded some effort from the children regarding motor skills like strength, coordination, endurance, and balance. In the physical skill training group, the requirements may have varied somewhat depending on the stations the children used more often. While children’s imagination was called for mainly in the yoga group, ball skills were trained only in the physical skill training group. Another important difference between the two groups is the fact that in the yoga group, the program was tied together by a story provided by the trainer, while in the physical skill training group, the different exercises were not related to each other. Finally, the physical skill training included more vigorous movements than the yoga training (e.g., bobsleigh run, riding a course on a large skateboard, Bobbycar^®^-race).

### Data Analysis

The aim of our pilot study was to investigate the influence of yoga practice as compared to physical skill training on motor function and physical self-concept as well as emotion and cognitive function. To this end we compared performance in a battery of tests and questionnaires before and after training in the experimental and comparison group.

We wondered whether there were differential changes in our dependent variables in the yoga and physical skill training group. To this end, we would have had to compute a two-way analysis of variance with group as between-subjects factor and session as within-subject factor. Since there was some variability in the pretest data, changes from pre- to posttest could probably vanish when analyzed across subjects. So we calculated differences between pre- and posttest values for each test and questionnaire (post-pre) and entered these differences into an analysis of covariance with group as between-subjects factor only and age as covariate. In addition, we asked whether the post-pre differences were statistically different from zero (one-sample *t*-tests). This was done to get an idea of how meaningful the changes from pre- to posttest were.

We conducted multivariate analyses instead of univariate analyses as it is plausible to assume that the scores belonging to one and the same test are mutually dependent. We report the general group effect of the multivariate analysis of covariance, as well as individual effects for each variable and the covariate’s effect.

Ahead of the multivariate analyses of covariance, for each group and variable, we determined outliers with the help of boxplots. We considered those trials as outliers, that lay outside (above and below) the whiskers, i.e., which were not within 1.5 time the interquartile range. For the Movement-ABC 2, 24 children entered the analyses, for the remaining tests and questionnaires, as said at the beginning, only 22 children were available.

Finally, the number of children included in the statistical analyses, their mean ages and the distribution of gender for the different tests and questionnaires applied are summarized in **Table 1**.

**Table 1:** Number of children included in the statistical analyses, their mean ages with standard errors and distribution of gender.

	Yoga group		Physical skill training group	
	Mean age/years (SE)	Number (gender)	Mean age/years (SE)	Number (gender)
PSC-C^1^	7.7 (0.3)	10 (7m,3f)/12	9.3 (0.5)	7 (4m,3f)/10
BAV 3–11^2^	7.7 (0.3)	10 (7m,3f)/12	9.8 (0.3)	8 (3m,5f)/10
M-ABC 2^3^	7.6 (0.3)	11 (7m,4f)/12	9.0 (0.5)	9 (3m,6f)/12
Flanker test	7.7 (0.4)	7 (4m,3f)/12	8.7 (0.6)	6 (2m,4f)/10
GoNogo test	7.7 (0.3)	10 (6m,4f)/12	9.1 (0.6)	9 (4m,5f)/10

^1^*PSC-C: physical self-concept for children;*^2^*BAV 3–11: anxiety questionnaire;*^3^*M-ABC 2: movement-ABC 2; m, male; f, female.*

## Results

Significant effects of group on post-pre differences were found for the PSC-C (category “speed”) and the BAV 3–11 (“problem-avoiding behavior” and “different strategies”). The remaining effects fell short of significance.

Since we tested children of different grades, preceding the actual statistical analysis we asked whether mean age was comparable between groups. A *t*-test for independent samples revealed that age differed significantly between groups (*t* = –2.809, *p* = 0.01). Therefore, in the subsequent statistical analyses, we used the children’s age as covariate.

Next, non-parametrical Chi-square tests were computed for the different tests and the yoga and physical skill training group to find out if there were significantly more boys/girls in each sample. In other words, we asked, for each test, whether there was a statistically significant imbalance of gender in the yoga or the physical skill training group. These tests revealed only non-significant results (*p* ≥ 0.206), which means that statistically, gender was equally distributed in both groups for each test. Thus, gender was not considered in the subsequent statistical analyses. The results are explained in detail in the following.

### PSC-C

**FIGURE 3:**
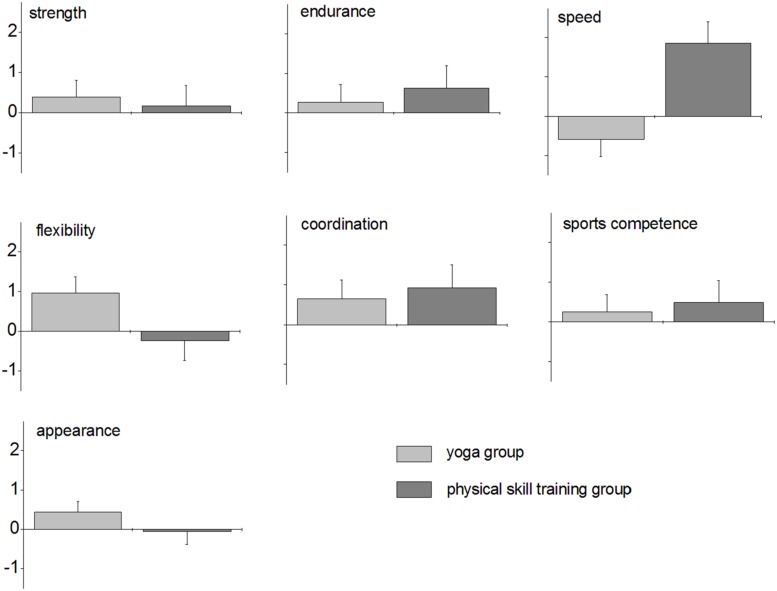
**Results of the PSC-C**. Age-corrected means and standard errors for the different categories of the PSC-C in the yoga group (light gray) and the physical skill training group (dark gray).

Age-corrected mean differences and standard errors of the mean for the different categories are illustrated in **Figure 3**. For each category, larger positive differences reflect improvements in the respective belief. This is because larger scores reflect stronger beliefs and we calculated the differences by subtracting the pre-values from the post-values.

It is evident from **Figure 3** that differences were generally small, i.e., ≤2 for both groups and all categories. Somewhat larger differences were found for the categories speed and coordination (physical skill training group) and flexibility (yoga group). *T*-tests for one sample revealed that post-pre differences were significantly different from zero in the yoga group for flexibility [*t*(9) = 2.753, *p* = 0.022]. None of the remaining post-pre-differences were significantly different from zero (yoga group: *p* ≥ 0.066, physical skill training group: *p* ≥ 0.172).

In the MANCOVA, group had no significant effect on dependent variables in general in the PSC-C [*F*_(7,8)_ = 1.612, *p* = 0.258, η^2^ = 0.585]. The same was true for the covariate age [*F*_(7,8)_ = 2.429, *p* = 0.119, η^2^ = 0.680]. **Figure 3**, however, shows that mean difference in speed was negative in the yoga group, whereas it was positive in the physical skill training groups. Thus, whilst children in the physical skill training group believed to be faster after the training (positive difference), this was not true for children in the yoga group, who reported to be less rapid than before (negative difference). The group difference was statistically significant [*F*_(1,14)_ = 10.146, *p* = 0.007, η^2^ = 0.420].

In the following, we go into some detail regarding individual results and changes. This is because our approach produces somewhat abstract results, i.e., group differences between post-pre differences, which might be difficult to interpret. Showing how many children revealed an increase/decrease in certain dependent variables may help to get a clear picture of the data. In the yoga group, 20% of the children (2 out of 10) revealed a positive difference, i.e., showed an increase in reported speed between pre- and post-test (70% no change, 10% decrease). In the sports group, 28.5% of the children (2 out of 7) revealed a positive difference (71.5% no change). Moreover, it has to be noted that there was one child in the physical skill training group with a comparably high positive difference of 4, while the other child had a difference of 1. In the yoga group, the maximum individual positive difference found was 1, the one child with a negative difference had a value of −2.

None of the remaining group differences were statistically significant [strength: *F*_(1,14)_ = 0.095, *p* = 0.762, η^2^ = 0.007; endurance: *F*_(1,14)_ = 0.200, *p* = 0.661, η^2^ = 0.014; flexibility: *F*_(1,14)_ = 2.812, *p* = 0.116, η^2^ = 0.167; coordination: *F*_(1,14)_ = 0.111, *p* = 0.744, η^2^ = 0.008; sports competence: *F*_(1,14)_ = 0.098, *p* = 0.759, η^2^ = 0.007; appearance: *F*_(1,14)_ = 1.034, *p* = 0.326, η^2^ = 0.069].

Finally, **Table 2** shows the mean pre- and posttest values of the seven categories of the PSC-C in the yoga and physical skill training group. It is evident that scores were generally high, respectively, close to maximum already in the pre-test.

**Table 2:** Means and standard errors in the pre- and posttest of the yoga group and the physical skill training group for the seven categories of the Physical Self-Concept for Children (PSC-C).

	Yoga group (*n* = 10)		Physical skill training group (*n* = 7)	
PSC-C	Pre	Post	Pre	Post
Strength (max. 12)	11.2 (0.4)	11.5 (0.2)	10.1 (0.7)	10.4 (0.4)
Endurance (max. 12)	10.5 (0.5)	10.9 (0.4)	10.4 (0.5)	10.9 (0.6)
Speed (max. 12)	11.6 (0.2)	11.6 (0.3)	10.3 (1.1)	11.3 (0.3)
Flexibility (max. 12)	10.7 (0.6)	11.5 (0.3)	10.3 (0.7)	10.3 (1.0)
Coordination (max. 12)	10.0 (0.6)	11.2 (0.4)	10.6 (0.5)	10.7 (0.3)
Sports competence (max. 24)	22.9 (0.3)	23.1 (0.5)	22.6 (0.4)	23.1 (0.4)
Appearance (max. 12)	11.1 (0.3)	11.4 (0.3)	10.3 (0.6)	10.4 (0.6)

*n, number of children included in the analysis; max, maximum score.*

### BAV 3–11

**FIGURE 4:**
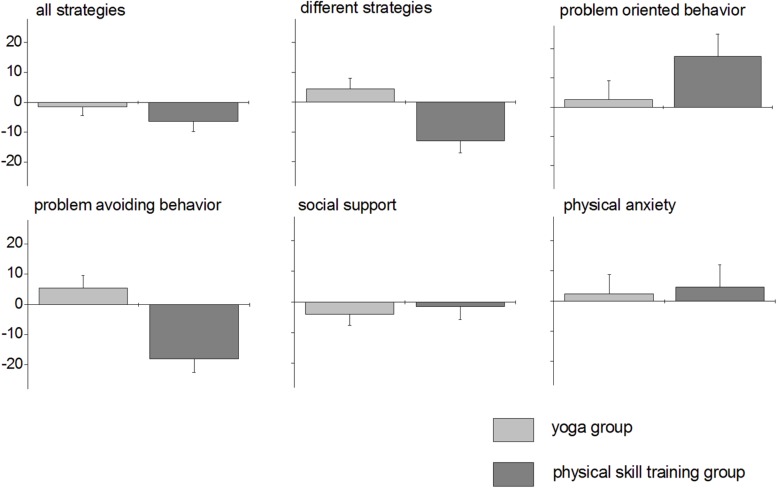
**Results of the BAV 3–11**. Age-corrected means and standard errors for the different sub scores of the BAV 3–11 in the yoga group (light gray) and the physical skill training group (dark gray).

**Figure 4** shows age-corrected mean differences between pre and posttest and standard errors of the mean for each group and variable. It shows that post-pre-differences were generally small (≤20), with somewhat larger differences in the physical skill training groups for the variables different strategies, problem-oriented behavior, and problem-avoiding behavior. *T*-tests for one sample revealed that post-pre differences were significantly different from zero in the physical skill training group for all strategies [*t*(7) = −2.413, *p* = 0.047]. None of the remaining post-pre-differences were significantly different from zero (yoga group: *p* ≥ 0.203, physical skill training group: *p* ≥ 0.061).

The multivariate analysis of covariance revealed a significant effect of group on dependent variables in general in the BAV 3–11 [*F*_(6,10)_ = 11.149, *p* = 0.001, η^2^ = 0.870]. The same was true for the covariate age [*F*_(6,10)_ = 10.599, *p* = 0.001, η^2^ = 0.864]. Mean post-pre-difference (averaged across subjects for each variable and across all variables) in the physical skill training group was −2.04 compared to 0.97 in the yoga group. Moreover, mean post-pre difference was −2.22 in older children (9–11) and 1.94 in younger children (6–8). (Please note that the age categories 6–8 years and 9–11 years were arbitrary chosen to illustrate the age effect on the mean difference. In the MANCOVA, age was entered as interval scaled variable.)

Moreover, significant group differences were found for problem-avoiding behavior [*F*_(1,15)_ = 10.933, *p* = 0.005, η^2^ = 0.422] and different strategies [*F*_(1,15)_ = 7.501, *p* = 0.015, η^2^ = 0.333].

The larger the problem-avoiding behavior-score is, the stronger the tendency to exhibit this kind of disadvantageous behavior. If yoga-training had a positive effect, we should find smaller values in the posttest than in the pretest, which would be evident in negative differences. In contrast to our expectations, a negative difference was found for the physical skill training group only, while there was a positive difference–even though small–in the yoga group.

In the yoga group, 5 out of 10 children showed a decrease in problem-avoiding score, in two children, values stayed the same, and in three children, there was an increase in problem-avoiding scores (50% – 20% – 30%). In the physical skill training group, four children out of eight revealed a decrease in problem-avoiding score; in four children, pre and post values were the same (50% – 50%).

The variable different strategies stands for the number of different coping strategies available to the child. The larger the score, the more divergent strategies are available. As we subtracted the prevalues from the postvalues, we would expect more positive differences in the yoga than in the physical skill training group. Figure 5 reveals that in both groups, less divergent strategies were available in the posttest than in the pretest, however, the effect was less pronounced in the yoga group.

Individual differences reflected the expected positive values in 2 out of 10 children (20%) in the yoga group, whereas five children revealed no change (50%) and three children a decrease in diversity of strategies (30%). In the physical skill training group, 1 out of 8 children showed a positive difference [12.5%; two children (25%): no change, five children (62.5%): decrease].

In addition, we found a trend to that effect, that fewer strategies were generally mentioned in the posttest compared to the pretest (variable all strategies; yoga group: −1.6, physical skill training group: −6.4). This effect was larger in the physical skill training group than in the yoga group. We asked whether this effect brought along the decrease in the variable different strategies. To this end, we correlated the variable all strategies with the variable different strategies in the post-test (with age as controlled variable) and found that this correlation was larger in the physical skill training group (*r* = 0.971, *p* < 0.0001) than in the yoga group (*r* = 0.687, *p* = 0.028).

The remaining effects of the MANCOVA were not significant: problem-oriented behavior [*F*_(1,15)_ = 1.568, *p* = 0.230, η^2^ = 0.095]; all strategies [*F*_(1,15)_ = 0.863, *p* = 0.368, η^2^ = 0.054]; social support [*F*_(1,15)_ = 0.134, *p* = 0.719, η^2^ = 0.009]; physical anxiety [*F*_(1,15)_ = 0.041, *p* = 0.843, η^2^ = 0.003].

**Table 3** shows the mean pre- and posttest *T*-values of the BAV 3–11 in the yoga and physical skill training group. It is evident that *T*-values lay in the normal range, i.e., between 35 and 65 in both groups and for all variables.

**Table 3:** Means and standard errors in the pre- and posttest of the yoga group and the physical skill training group for the sub-scores and the total score of the anxiety questionnaire (BAV 3–11).

	Yoga group (*n* = 10)		Physical skill training group (*n* = 8)	
BAV 3–11	Pre	Post	Pre	Post
All strategies	37.6 (2.28)	35.8 (1.36)	40.0 (3.28)	33.9 (1.48)
Different strategies	40.2 (3.66)	40.4 (4.30)	40.6 (3.51)	33.0 (2.55)
Problem-oriented behavior	43.0 (4.77)	51.2 (3.36)	43.8 (7.22)	54.1 (8.98)
Problem-avoiding behavior	58.4 (5.13)	56.1 (3.87)	43.8 (5.15)	35.5 (1.55)
Social support	45.6 (3.26)	44.8 (2.66)	45.1 (2.77)	39.9 (0.74)
Physical anxiety	58.5 (5.17)	60.8 (5.56)	45.3 (3.81)	49.9 (5.13)
Total score	38.7 (2.41)	38.7 (2.01)	41.6 (3.23)	39.0 (2.75)

*n, number of children included in the analysis.*

### Correlation Between the Physical Self-Concept and Anxiety

Kaley-Isley et al. (2010) argue that increasing self-awareness, especially of limitations while learning a new skill like yoga, may lower self-esteem. Post-hoc, we therefore asked whether negative changes in self-concept may impair the handling of problems, especially due to avoidance-behavior. To test this additional hypothesis, we computed correlations between post-pre differences of a total PSC-C score (total sum) with post-pre differences of the BAV 3–11 problem-avoiding score. The results were as follows: physical skill training group (*df* = 3) *r* = −0.942, *p* = 0.017, yoga group (*df* = 6) *r* = −0.053, *p* = 0.901, complete group (*df* = 12) *r* = −0.346, *p* = 0.226. Thus in the physical skill training group, children with larger increases in total PSC-C score revealed larger decreases in the BAV 3–11 problem-avoiding score. Otherwise stated, the larger the decreases in PSC-C score, the larger the increases in BAV 3–11 problem-avoiding score.

### Executive Functions and Movement-ABC 2

#### Executive Functions

All in all, group had no significant effect on dependent variables in the Flanker–Test [*F*_(6,5)_ = 2.774, *p* = 0.141, η^2^ = 0.769]. The same was true for the covariate age [*F*_(6.5)_ = 0.614, *p* = 0.716, η^2^ = 0.424]. Individual group effects on reactions times (compatible and incompatible stimuli with reactions on the left and right) and errors (compatible and incompatible) fell short of significance (*p* ≥ 0.082).

**Table 4** shows the mean pre- and posttest values of the Flanker test in the yoga and physical skill training group. It is evident that error rates were generally small. Reaction times were slightly higher in the yoga group than in the physical skill training group and in the incompatible conditions compared to the compatible conditions. Furthermore, reaction times decreased slightly from pre- to posttest in both groups. *T*-tests for one sample revealed that post-pre differences were significantly different from zero in the incompatible condition on the right [yoga group: *t*(6) = −2.884, *p* = 0.028, physical skill training group *t*(5) = −2.886, *p* = 0.034]. None of the remaining post-pre-differences were significantly different from zero (yoga group: *p* ≥ 0.072, physical skill training group *p* ≥ 0.076).

**Table 4:** Means and standard errors in the pre- and posttest of the yoga group and the physical skill training group for the different conditions of the Flanker test.

	Yoga group (*n* = 7)		Physical skill training group (*n* = 6)	
Flankertest	Pre	Post	Pre	Post
Reaction time [ms]	841.3	767.4	714.6	740.0
compatible condition right	(118.2)	(77.0)	(82.2)	(50.3)
Reaction time [ms]	1084.2	740.4	902.2	722.5
incompatible condition right	(167.7)	(65.2)	(106.4)	(63.4)
Reaction time [ms]	805.3	800.3	1019.2	705.6
compatible condition left	(107.6)	(90.8)	(223.7)	(57.5)
Reaction time [ms]	898.4	770.9	920.6	723.0
incompatible condition left	(138.4)	(102.7)	(144.9)	(57.5)
Total	907.3	769.7	889.1	722.8
reaction time [ms]	(129.8)	(74.0)	(129.3)	(50.5)
# errors compatible condition	0	0	0.5	0
	(0)	(0)	(0.2)	(0)
# errors incompatible condition	0.1	0	0.2	0
	(0.1)	(0)	(0.2)	(0)

*n, number of children included in the analysis.*

Moreover, group had no significant effect on dependent variables in general in the Go-Nogo-Test [*F*_(3,14)_ = 0.009, *p* = 0.999, η^2^ = 0.002]. The same was true for the covariate age [*F*_(3,14)_ = 0.697, *p* = 0.569, η^2^ = 0.130]. Individual effects of group on total reaction time and number of false errors and misses were also not significant (*p* ≥ 0.865).

**Table 5** shows the mean pre- and posttest values of the Go-Nogo test in the yoga and physical skill training group. It is evident that error rates were generally small. Moreover, reaction times were slightly higher in the yoga group than in the physical skill training group and decreased slightly from pre- to posttest in both groups. *T*-Tests for one sample revealed that none of the post-pre-differences were significantly different from zero (yoga group: *p* ≥ 0.150, physical skill training group *p* ≥ 0.397).

**Table 5:** Means and standard errors in the pre- and posttest of the yoga group and the physical skill training group for the reaction times [ms] and number of errors in the Go-Nogo test.

	Yoga group (*n* = 10)		Physical skill training group (*n* = 9)	
Go-Nogo test	Pre	Post	Pre	Post
Reaction time [ms]	517.2 (26.1)	497.0 (20.0)	467.3 (25.0)	463.2 (19.6)
# errors (false reaction)	2.6 (0.6)	1.6 (0.5)	2.0 (0.7)	1.3 (0.5)
# errors (omissions)	0.9 (0.2)	0.6 (0.4)	0.4 (0.2)	0.4 (0.2)
# errors (total)	3.5 (0.6)	2.2 (0.6)	2.4 (0.6)	1.8 (0.5)

*n, number of children included in the analysis.*

#### Movement-ABC 2

All in all, group had no significant effect on dependent variables in the Movement-ABC 2 [*F*_(3,15)_ = 0.241, *p* = 0.866, η^2^ = 0.046]. The same was true for the covariate age [*F*_(3,15)_ = 0.158, *p* = 0.923, η^2^ = 0.031]. Individual group effects of the multivariate analysis of covariance on hand skills, ball skills, and balance were not significant (*p* ≥ 0.534).

**Table 6** shows the mean pre- and posttest values of the Movement-ABC 2 in the yoga and physical skill training group. It is evident that the means lay in the medium range (scores between 10 and 12 correspond with percent ranks 50 and 75). Moreover, scores were slightly higher in the yoga group than in the physical skill training group and did not increase from pre to post test. Accordingly, *t*-tests for one sample revealed that none of the post-pre-differences were significantly different from zero (yoga group: *p* ≥ 0.108, physical skill training group: *p* ≥ 0.301).

**Table 6:** Means and standard errors in the pre- and posttest of the yoga group and the physical skill training group for the different scores of the Movement-ABC 2.

	Yoga group (*n* = 11)		Physical skill training group (*n* = 9)	
M-ABC 2	Pre	Post	Pre	Post
Hand skills (max. 19)	10.4 (1.0)	10.9 (1.0)	11.4 (0.4)	10.7 (0.6)
Ball skills (max. 19)	12.6 (0.8)	12.0 (0.5)	10.8 (0.9)	10.1 (0.5)
Balance (max. 19)	12.5 (0.5)	13.9 (0.4)	12.1 (0.8)	12.4 (0.8)
Total (max. 19)	12.6 (0.8)	12.7 (0.7)	11.9 (0.4)	11.4 (0.5)

*n, number of children included in the analysis; max, maximum score.*

## Discussion

In the present pilot study we investigated the effects of yoga vs. physical skill training in primary school-aged children on motor functions and physical self-concept, as well as emotional and cognitive functions. Significant differences between the yoga group and the physical skill training group in post-pre differences were found in the category speed of the physical self-concept for children (PSC-C) and the variables all strategies and problem-avoiding behavior of the anxiety questionnaire (BAV 3–11). With respect to executive function and motor skills, no differences between groups were found.

Mean perceived speed in the PSC-C decreased in the yoga group, while it increased in the physical skill training group (mean negative vs. mean positive post-pre differences). With this result, one has to take into account that it was only one child in the yoga group (10%), which actually reported a deceleration (vs. 0% in the physical skill training group). Moreover, in the yoga group, 20% of the children showed an increase in perceived speed, while it was 28.5% in the physical skill training group. Moreover, the significant group effect may be put into perspective by the observation that there was one child in the physical skill training group with a comparably large positive difference, which enhanced the mean value in this group.

Nevertheless, a training which concentrates on certain positions (asanas) and meditative elements should be more prone to lead to a perceived (and maybe actual) deceleration than physical skill training. As mentioned in the methods section, the physical skill training included more vigorous movements than the yoga training, which may bring about such effects. This is a research question worthwhile investigating in more detail in future studies. Generally, it appears helpful to more closely look at effects, which are specific to the kind of movement: for example, in the present study, post-pre differences in the subscale flexibility were significantly different from zero in the yoga group only. This result is in accordance with the idea that yoga is about the mastery of certain (more or less difficult) asanas, which put greater demands on flexibility than sports in general. It appears that the children in the yoga group–more than children in the physical skill training group–received the impression to be more flexible after the training than before. Put the other way around, it might be too unspecific to just look at the broader concept of self-esteem instead of physical self and its facets. Accordingly, Lindwall et al. (2014) found that changes in physical activity were related to changes in physical self-perception, but not in global self-esteem in adolescent girls. With respect to the level of cognitive development, it appears reasonable to assume that the self-perception of children is not as multidimensional and does not have the same hierarchical structure as that of older children or adults (Case, 1991). As this training addressed mostly the physical domain, effects would be expected on corresponding facets of the physical self, but not on facets on a higher, more abstract level of self-perception (Sonstroem and Morgan, 1989).

Finally, small to missing effects of yoga vs. sport activities on other categories of perceived self-concept may trace back to ceiling effects. Children’s self-perceptions were close to maximum already with the pretest in many subjects. Thus, in future studies with healthy primary school-aged children, one should especially pay attention to initially high values. Moreover, an age-dependent tendency to evaluate the own skills as too positive may also have played a role. Existing literature suggests that the evaluation of the own physical skills gets more accurate with increasing age (Schmidt et al., 2013). Especially at the beginning of the primary school, descriptions of the self are still largely positive (Harter, 2006).

Next, mean difference in the variable different strategies of the BAV 3–11 was negative in the physical skill training group, while it was positive in the yoga group. This effect was somewhat more robust than the above-mentioned effects in the PSC-C speed subscale, as 62.5% of the children in the physical skill training group revealed a decrease in diversity of strategies as compared to 30% in the yoga group (increase: 20% yoga group, 12.5% physical skill training group).

In addition, we found a trend to that effect, that fewer strategies were generally mentioned in the post-test compared to the pre-test (variable all strategies). This effect was larger in the physical skill training group than in the yoga group, and the post-pre difference was statistically different from zero only in the physical skill training group. Moreover, the variable all strategies was significantly correlated with the variable different strategies in the posttest and this correlation was larger in the physical skill training group than in the yoga group.

Thus, children in the physical skill training group may have had a somewhat larger tendency than children in the yoga group to name fewer strategies in the posttest than in the pretest and this tendency brought about the naming of fewer different strategies. One may only speculate about the underlying reasons. Maybe unspecific effects like the experimenter or time of day or date may play a role. It has to be kept in mind that the posttest was performed at the end of the term just before summer break.

Mean post-pre difference in the problem-avoiding score was positive in the yoga-group, while it was negative in the physical skill training group. Although in the physical skill training group, the comparably large mean difference was primarily due to one child, a possible interpretation of this result–which could be investigated in future studies–is the following: awareness (for the self, but also for the world) may have increased in the children of the yoga group. This may be an effect of the yoga training which encouraged the boys and girls to look inside his- or herself and follow the trainer’s mental journal while doing yoga poses. Actually, this increase in awareness would be the aim of yoga training; however, a raise in awareness may generalize to all aspects of body and life, both positive and negative. Given that no coping strategies were taught, some problematic situations may then seem less easily to cope with. In other words, seeing a problem more clearly than before, without an increased feeling of competence in handling this problem, may lead to an avoidance strategy. In an analogous fashion, White (2012) concludes: “[…] it is possible that the increasing awareness of stressors in itself increased stress, possibly as part of the process of developing mindfulness or related to cognitive, emotional, or social development. Mindfulness in children may differ from mindfulness in adults and warrants further investigation.”

The association between increased self-awareness and anxiety may be related to self-esteem. Kaley-Isley et al. (2010) argue that increasing self-awareness may lower self-esteem and increase existing anxiety when learning something new at which a person is not skilled. According to this hypothesis, in a study by Benavides and Caballero (2009) a decrease in self-concept was found after yoga training. Maybe, in the present study, changes in the BAV 3–11 problem-avoiding score were also related to self-awareness. Kaley-Isley et al. (2010) further argue that children with anxiety benefit from more physically active forms of yoga, at least initially, in order to shift their attention away from mental preoccupations. Thus, more active forms of physical activity–as was practiced in the physical skill training –may avoid this effect.

Finally, the BAV 3–11 was the only test showing a general effect of group and age on the dependent variables. In the physical skill training group, mean difference was negative, while in the yoga group, it was positive. These mean differences across variables are somewhat difficult to explain, however, because in some cases, a positive difference meant an improvement in anxiety (all strategies, different strategies, problem-oriented behavior, social support), while in others, it described degradation (problem-avoiding behavior, physical anxiety). Nevertheless, on the average, children in the physical skill training group produced smaller *T*-values in the post test compared to the pretest, while the effect was the other way around in the yoga group. In addition, mean difference was negative in older children and positive in younger children. Thus, the group effect may rely on the older children who were overrepresented in the physical skill training group. In sum, there were no strong effects of yoga or physical skill training on the anxiety questionnaire, which may be also due to the fact that scores were generally in the normal range.

With respect to executive function, missing group differences both with Flanker and Go-Nogo tests may be due to the fact that the tasks were too simple. Very small error rates argue in favor of this interpretation. The reaction times were in a range to be expected from children of that age (Yang et al., 2011; Brydges et al., 2014). In addition, in the Flanker test we found that only the post-pre difference of reaction time in the incompatible condition on the right significantly differed from zero. Maybe this condition was more prone to training effects, as it was a more difficult condition with initially higher reaction times. However, it is not quite clear why the incompatible condition on the left should be easier – even more, as the right side is the side of the dominant hand. However, all in all, practice effects were more pronounced in the incompatible compared to the compatible condition in the Flanker test in the present study (compare Rueda et al., 2004).

The negative effects are in accordance with the conclusions of Diamond and Lee (2011), who reviewed the efficacy of different training methods to improve executive functions. They resume that improvements in executive functions are most evident when executive function demands are greatest. As with the PSC-C, beneficial effects of yoga or physical skill training may be hard to find in case maximum or at least age-appropriate performance is given right from the start. Thus, regarding future studies, one should resort to more demanding tests. Accordingly, in the study by Gothe et al. (2013), effects of yoga training as compared to aerobic exercise and a baseline condition were found for more difficult task variations with greater demands on executive control. In the latter study, acute effects of yoga were investigated, which means that subjects were presented with the executive tasks immediately after the training. Thus, effects in the present study may have been stronger with shorter time spans between training and test session. Nevertheless, another possible reason for the missing effects would of course be lacking power, as the number of subjects included in the multivariate analysis of covariance was small.

Small mean post-pre differences at least in hand skills of the M-ABC 2 were surprising given the beneficial effects of yoga on hand skills in the study of Telles et al. (1993) with a quite similar task. Maybe differences in duration or intensity of yoga training may account for these controversial results. In the study of Telles et al. (1993), children took part in a 10-days yoga training period, i.e., with training at ten subsequent days, which was probably more effective than our 12 training sessions distributed among 6 weeks. Moreover, within groups pre-post comparisons were performed instead of MANCOVAS, i.e., differences in statistical approaches may also play a role.

One may also argue that we did not find differences between groups because the skills that were trained in the yoga group and the physical skills training group were quite similar. However, post-pre differences were generally small, i.e., there was no real change from pre to post test (not an equally large change in both groups).

A general shortcoming of the present study was of course the small sample size. As a consequence, we might have been unable to reveal differences between groups due to too large a variability within groups. Small sample-sizes, comparably large intra-group variabilities and unequal sample sizes of the two groups may have reduced the power of our statistical tests, an assumption which was supported by post-hoc power analyses we performed with G^∗^Power (http://www.gpower.hhu.de/; Faul et al., 2007). The 1-ß-error probability varied between 0.2556 for the Flanker-Test and 0.7491 for the GoNogo-Test (PSC-C: 0.3725, BAV 3–11: 0.4817, M-ABC: 0.7053). Moreover, the age range investigated was quite large and we found significant differences in mean age between the yoga and the physical skill training group. Ideally, mean age should be comparable between the two groups, because age probably has an influence on test results as well as on the changes between pre- and posttests. Thus, if mean age substantially differs between groups, it is difficult to trace effects on the dependent variables back to the different trainings. To account for this problem, we included age as a covariate in the statistical analyses. The same problem affects the variable gender. The number of girls and boys each differed between the yoga and the physical skill training group (respectively, between the two samples that were actually used for each statistical test). However, non-parametrical Chi-square-tests revealed no statistically significant imbalances in gender distributions in neither group, so we did not consider gender as a covariate. Nevertheless, one cannot exclude that the slight actual gender imbalances had an impact on our results, which represents, next to age differences, a further shortcoming of our study.

When thinking about directions and recommendations for future research, the following aspects may be payed attention to. Problems with healthy children specifically comprise the fact that performance lies in the normal range. Questionnaires have to leave enough scope to reflect variations in this range, i.e., they have to be quite sensitive. Moreover, with respect to executive functions, it would be advisable to resort to more demanding tests to really challenge the children, so that improvements due to training can be observed in the first place. Otherwise speaking, the problem of ceiling effect may be reduced by using more difficult tasks. This is feasible in cognitive tests, but with non-cognitive measures of anxiety or self-concept, other solutions have to be found. Related to the problem of ceiling effects, and maybe somewhat specific to the notion of (physical) self-concept, is the advisement that developmental changes have to be taken into account. (We argued the ceiling effects in the physical self-concept may have been due to the fact that primary school-aged children tend to overestimate their physical skills for developmental reasons). Second, and also related to the development of the physical self-concept, is that the categories measured (e.g., flexibility) should be meaningful with respect to the kind of training performed and not be on an abstract level. This is because the self-perception of children is not as multidimensional and does not have the same hierarchical structure as that of older children or adults. Third, in the present study it is assumed that problem-avoiding behavior after yoga training may be correlated with changes in the self-awareness, which may be investigated in future studies. This may be an important proposition, as it points to the necessity to consider complex interactions between self-concept, anxiety, attention/awareness, and the stage of learning. It also shows that it is probably not sufficient to show children how to increase awareness, but they must also be equipped with some coping-strategies. Thus, in future studies, it would be advisable to use longer training phases with concomitant assessment of self-concept and self-awareness not only before and after, but also during training. In addition, to prevent adverse effects of yoga training–which needs more attention in research anyway–, emphasis should be put on coping-strategies in parallel.

## Conclusion

In sum, we found only single significant differences between the yoga group and the physical skill training group in an array of tests of motor functions and physical self-concept, anxiety, and executive function. This may be due to the fact that we investigated healthy children, that sample size was small and that the age-range investigated was large. There was a decrease in perceived speed in the yoga group, while taking part in physical skill training led to an increase in perceived speed. We showed that this result has to be taken with caution; however, one reason for this effect might be that yoga puts an emphasis on slow – or at least not hectic – movements. Additionally, more problem-avoiding behavior after yoga training may be correlated with changes in the self-awareness during different learning stages in the yoga training. The advantage of this study lies in the investigation of different aspects of the impact of yoga, i.e., body, emotion and cognition. However, more studies with a bigger sample size have to be conducted.

## Author Contributions

SR: conception of the work; acquisition, analysis, and interpretation of data; drafting the work or revising it critically; final approval of the version to be published; agreement to be accountable for all aspects of the work. MT: conception of the work; acquisition, analysis, and interpretation of data; drafting the work or revising it critically; final approval of the version to be published; agreement to be accountable for all aspects of the work. SZ: acquisition, analysis, and interpretation of data; drafting the work or revising it critically; final approval of the version to be published; agreement to be accountable for all aspects of the work. SQ: acquisition, analysis, and interpretation of data; drafting the work or revising it critically; final approval of the version to be published; agreement to be accountable for all aspects of the work. PJ: conception of the work; acquisition, analysis, and interpretation of data; drafting the work or revising it critically; final approval of the version to be published; agreement to be accountable for all aspects of the work.

## Conflict of Interest Statement

The authors declare that the research was conducted in the absence of any commercial or financial relationships that could be construed as a potential conflict of interest.
